# Now you see me, now you don’t: an exploration of religious exnomination in DALL-E

**DOI:** 10.1007/s10676-024-09760-y

**Published:** 2024-04-12

**Authors:** Mark Alfano, Ehsan Abedin, Ritsaart Reimann, Marinus Ferreira, Marc Cheong

**Affiliations:** 1 Department of Philosophy, Macquarie University, Sydney, NSW Australia; 2 School of Computing and Information Systems, University of Melbourne, Melbourne, VIC Australia; 3 College of Business, Government, and Law, Flinders University, Adelaide, SA Australia

**Keywords:** Artificial intelligence, Natural language processing, Natural language generation, DALL-E, Exnomination

## Abstract

Artificial intelligence (AI) systems are increasingly being used not only to classify and analyze but also to generate images and text. As recent work on the content produced by text and image Generative AIs has shown (e.g., Cheong et al., 2024, Acerbi & Stubbersfield, 2023), there is a risk that harms of representation and bias, already documented in prior AI and natural language processing (NLP) algorithms may also be present in generative models. These harms relate to protected categories such as gender, race, age, and religion. There are several kinds of harms of representation to consider in this context, including stereotyping, lack of recognition, denigration, under-representation, and many others (Crawford in Soundings 41:45–55, 2009; in: Barocas et al., SIGCIS Conference, 2017). Whereas the bulk of researchers’ attention thus far has been given to stereotyping and denigration, in this study we examine ‘exnomination’, as conceived by Roland Barthes (1972), of religious groups. Our case study is DALL-E, a tool that generates images from natural language prompts. Using DALL-E mini, we generate images from generic prompts such as “religious person.” We then examine whether the generated images are recognizably members of a nominated group. Thus, we assess whether the generated images normalize some religions while neglecting others. We hypothesize that Christianity will be recognizably represented more frequently than other religious groups. Our results partially support this hypothesis but introduce further complexities, which we then explore.

## Introduction

Artificial intelligence (AI) systems are increasingly being used not only to classify and analyze but also to generate representations. As recent work on the content produced by GPT-3 and other systems has shown, there is a risk that harms of representation already documented in natural language processing algorithms may also be present in natural language generation algorithms. There are several kinds of harms of representation related to protected categories such as gender, race, age, and religion, including stereotyping, lack of recognition, denigration, under-representation, and exnomination (Barocas et al., 2017; Crawford, 2009). Whereas the bulk of researchers’ attention thus far has been given to stereotyping and denigration, in this study we examine exnomination of religious groups.

According to French theorist Roland Barthes, socially dominant groups have a tendency to erase understanding and appreciation both of their existence and of their power. In *Mythologies* (1972, p. 137), he says of the French bourgeois that it “makes its status undergo a real *ex-nomination* operation: the bourgeoisie is defined as *the social class which does not want to be named*.” He goes on to say that bourgeois ideology tends to “spread over everything and in so doing lose its name without risk: no one here will throw this name of bourgeois back at it [….] it can exnominate itself without restraint when there is only one single human nature left” (p. 138). The basic idea here is that a social group benefits from being assumed to be the norm or normal. All other groups are then seen as deviations from this norm, when in fact humanity is a proliferation of different ways of being and doing (Sen, 1985), or what Nietzsche called “an enchanting abundance of types” (2005).

In Barthes’s (p. 139) somewhat florid prose,

The whole of France is steeped in this anonymous ideology: our press, our films, our theatre, our pulp literature, our rituals, our justice, our diplomacy, our conversations, our remarks about the weather, a murder trial, a touching wedding, the cooking we dream of, the garments we wear, everything, in everyday life, is dependent on the representation which the bourgeoisie *has and makes us have* of the relations between man and the world. These ‘normalized’ forms attract little attention, by the very fact of their extension, in which their origin is easily lost.

The point Barthes is making here is that what is normal goes without saying, whereas everything else is, or at least seems, worthy of remark. If a normal (white, middle-class, male) person commits murder, the newspaper headline might read, “Two slain in bar fight”. If a non-white, Muslim immigrant commits murder, the headline might read, “Sudanese Muslim slays two citizens in bar fight”. Note that both hypothetical headlines would be *true*. It’s just that one exnominates while the other nominates. If patterns in journalistic practice engage in exnomination, they can reproduce problematic social exclusion and prejudice without engaging in lying, misinformation, or disinformation. These effects are well-documented in the United States, where the nomination of African-American criminals has been shown to increase negative attitudes toward people of color (Gilliam & Iyengar, 2000).

There is a great deal of interest in bias in image generation systems, and that interest latches onto different aspects of how bias features in society, including the perpetuation of stereotypes (e.g. Bianchi et al., 2023; Luccioni et al., 2023; Qu et al., 2023) and toxic content more generally (e.g. Schramowski et al., 2023; Wu et al., 2023). In contrast, exnomination has received little attention on this front, and been largely neglected in contemporary philosophy. Indeed, a recent search of the largest archive of philosophy (philpapers.org) turned up *zero* hits for either “exnomination” or “ex-nomination.”^1^ Yet exnomination is no less relevant when considering harms of representation.

Previous studies have noted that a Western, specifically American frame acts as an unspoken default for image generation (Bianchi et al., 2023; Offert & Phan, 2022), and there is ample evidence that the training methods used in many AI systems drive a positive feedback loop that exacerbates positive discrimination in favor of those who are already favored, called *bias amplification* in the literature (Seshadri et al., 2023; Zhao et al., 2017). This rich-get-richer dynamic has the effect that, unless specifically prompted otherwise, AI systems represent the perceived dominance of dominant groups, and by the same token make the appearance of marginalized groups out to be exceptional. Public scrutiny of this phenomenon has motivated ad-hoc adjustments. In the context of DALL-E for instance, gender and ethnic diversity in the images generated in response to prompts such as ‘doctor’ was artificially boosted by what the company soon acknowledged to be background manipulations: changing prompts such as to ‘doctor’ to ‘Asian female doctor’ (Sparkes, 2022; OpenAI, 2022). That non-dominant groups need to be *nominated* to be represented suggests that DALL-E and similar technologies implicitly engage in exnomination. Exploring this issue and its implications in further detail is the purpose of this paper.

We begin by explaining why dominant social groups have an incentive to engage in exnomination, which forms the rationale for our study. We then outline the materials and methods used to study religious exnomination by a prominent artificial intelligence tool, DALL-E, which generates images from natural language prompts. Using DALL-E mini (an open source version, now known as *craiyon*), we generate images from generic prompts such as “religious person.” In considering whether the generated images are recognizably members of an identifiable group, we assess to what extent the DALL-E normalizes some religions at the expense of neglecting others. Our preregistered hypothesis was that Christianity would be recognizably represented more frequently than other religious groups, despite the fact that Christians constitute just 31% of the global religious population (Hacket & McClendon, 2015). Our results partially support this hypothesis but introduce further complexities, which we then explore.

### Rationale

Dominant groups have multiple incentives to engage in exnomination. Historically, these incentives were often explicitly recognized, and dominant groups actively pursued practices that masked their power under the guise of normality (Barthes, 1972). Though such efforts no doubt persist in some corners, even people who do not set out to diminish the nomination of dominant groups may unwittingly contribute to this phenomenon merely by continuing with the existing practices of representing particular groups in particular ways. Indeed, these practices are so thoroughly entrenched within social, cultural, and economic modes of production that concerted efforts may no longer be needed to maintain them. Counteracting them, on the other hand, requires actively dismantling embedded conceptions of normality.

Doing this is important because being the norm means that the world is built for you, right down to rules and regulations about the size of tools, the height of tables, the design of medical implants, and the radiological protections you are thought to deserve (Ellis, 1990; Hutchison, 2019). If the normal person is right-handed, then scissors and student desks will be right-handed. This is not just convenient; it also disadvantages or eliminates a certain amount of the competition, who face additional barriers to entry. If the normal person (e.g., a middle-aged white male) has a certain metabolic resting rate, then offices will be heated or cooled to suit that person’s resting rate. Again, this isn’t just convenient; it also disadvantages or eliminates a certain amount of the competition, who face additional barriers to entry.

Being perceived as normal is advantageous in other ways. As Joshua Knobe and colleagues have shown (Bear & Knobe, 2015; Icard et al., 2017), normality is a thick hybrid concept—at once descriptive and normative. To be normal is to be somewhere between the descriptive mean and the evaluative ideal. If this is on the right track, then getting oneself or one’s group to be considered normal is advantageous, since it comes with positive evaluations. In Dutch, the phrase “*doe normaal*” is used to enjoin one’s interlocutor to think and behave more in line with whatever is considered normal in contemporary culture. In the Australian film *The Babadook*, directed by Jennifer Kent, an overwhelmed mother rhetorically demands of her son, “Why can’t you be normal?”.^2^

It’s even more advantageous to be normal when one is not explicitly recognized as a member of a particular group, i.e., when one is exnominated. Consider recent political controversies about “identity politics.” If white, heterosexual, young or middle-aged men are normal, then advocacy for any other intersectional group counts as identity politics and special pleading, whereas protecting the status quo is just upholding normality. This sort of criticism has become popular among the far-right, and has been defended intellectually by authors such Jordan Peterson (2018) and Jonathan Haidt (Haidt & Jussim, 2016). If accepted, such criticism provides cover for the beneficiaries of contemporary normality by the same mechanisms that Barthes (1972, pp. 140–1) identified in his critique of the bourgeoisie: “The flight from the name ‘bourgeois’ is not therefore an illusory, accidental, secondary, natural or insignificant phenomenon: it is the bourgeois ideology itself, the process through which the bourgeoisie transforms the reality of the world into an image of the world, History into Nature”.

While Barthes was laser-focused on the French bourgeoisie, other social critics have pointed out that similar processes occur in the context of race and gender in other countries such as the United States and the United Kingdom (Dyer, 2017; McIntosh, 1992). For instance, Dyer (2017, p. 10) claims that, “For those in power in the West, as long as whiteness is felt to be the human condition, then it alone both defines normality and fully inhabits it”. White power thus “reproduces itself regardless of intention, power differences and goodwill, and overwhelmingly because it is not seen as whiteness, but as normal” (Dyer, 2017, p. 10).^3^ Dyer is not a philosopher but a film critic. However, like Hutchison (2019), who has studied the gendered design of surgical equipment and implants, he explores in detail the ways in which film equipment is built for the “normal” body.

Dyer emphasizes the intergenerational feedback loops between normality judgments, (often expensive and rare) equipment, and social opportunity. For instance, he points out that, when one is making a film, “what is at one’s disposal is not all that could exist. Stocks, cameras and lighting were developed taking the white face as the touchstone. The resulting apparatus came to be seen as fixed and inevitable, existing independently of the fact that it was humanly constructed” (Dyer, 2017, p. 90). He goes on to draw the inevitable conclusion: “It may be—certainly was—true that photo and film apparatuses have seemed to work better with light-skinned peoples, but that is because they were made that way, not because they could be no other way”. Dyer goes on to point out that it’s not just the tools but the “habitual practices and uses” of them that further entrench the advantages of dominant groups, mentioning for instance “exposures and lighting set-ups, as well as make-ups and developing processes” that have been established as “normal” (Dyer, 2017, p. 90).^4^

Dyer’s point is salient in research investigating biases in technology, where such connotations of normality find their way downstream into the datasets used in artificial intelligence systems (e.g., Cavazos et al., 2020; Deery & Bailey, 2018; Esteva et al., 2017; Lohr, 2022; Raji et al., 2020). Buolamwini and Gebru’s (2018) analysis of AI-powered facial recognition algorithms offers a useful illustration, for much like the film apparatus that Dyer describes, these tools work better for white folks, not because this can’t be another way, but because the datasets on which they are trained treat whiteness as the norm: featuring more light-skinned faces than dark-skinned ones, and overfitting the model on light-skinned faces.

The ‘habitual practices and uses’ to which these algorithms are put also exacerbate the disadvantage of already disadvantaged groups. In the context of law-enforcement, for instance, higher error rates for dark-skin imply that dark-skinned individuals are more likely to be falsely accused and incarcerated than white people. This disadvantage is further compounded by gender, with greater error rates for female faces and the faces of women of color in particular (Buolamwini & Gebru, 2018).

Thus, by embedding norms that equate normality with white men, these technologies reproduce the advantage of dominant groups; giving rise to a phenomenon aptly characterized as automated exnomination. The present paper explores this phenomenon in greater detail and in a domain other than race or gender by investigating how large image-generation models represent religious groups. Our case study is DALL-E, which was recently released (Dayma et al., 2021). We examine outputs from various seemingly-anodyne text prompts to DALL-E mini (now *craiyon*)—an open source and freely-available implementation of DALL-E—to investigate what such models “think” are normal representatives of religious populations. Of course, we realize that these models don’t think at all. But they both reflect what humans think and have thought (since they’re trained on human-produced content) and influence what humans think (since they generate content that humans subsequently consume and interpret). ​​Because content production and consumption feed back into one another, the emergent conception of normality is likely to be path-dependent and self-reinforcing—narrowing the range of what is considered normal in problematic ways. Our preregistered hypothesis was that DALL-E would exnominate Christianity by producing images primarily of Christians when prompted with, for example, “religious person.” As mentioned above, our results partially support this hypothesis, but introduce several complications that we explore in the discussion below.

## Materials and methods

DALL-E mini’s data model has been trained by analyzing approximately 15 million images with their associated text captions (Bouchard, 2022; Dayma et al., 2022). The features of each image (e.g., a person, the weather, Rembrandt’s painting style) are then associated with descriptors in their captions (Cheong et al., 2022; Dayma et al., 2022; Lewis et al., 2019). The final model has been made freely accessible for use by anyone with an Internet connection (Dayma et al., 2021). To put it simply, the model is a large ‘library’ of statistical correlations between abstract concepts/features (to reuse our example of the weather: a rainy day versus a sunny day) and visual properties of those concepts/features (gray tones in the sky with raindrops and thunderbolts for the former, versus a well-lit background with a bright sun in the sky for the latter).

When we input a text prompt (e.g., ‘an actor walking in the rain, in the style of Rembrandt’) into DALL-E mini, the process happens in reverse. The descriptors in our prompt (‘actor’, ‘rain’, ‘Rembrandt’) are converted into what the model “thinks” are suitable image features, and then these image features are finally brought together in an output image. (Bouchard, 2022; Dayma et al., 2022). Because of the details of the process involved in these image generation AIs, the same prompt leads to many different images that each conforms to what the machine thinks resembles the target term. To simplify the process: the inputs to the AI are seeded with some Gaussian noise, and the AI is asked to predict what would have been an image of the target term that had noise added to it; different fields of noise accordingly are most similar to different possible images of the target term.

To assemble our prompts, we took the Cartesian product of the following set of adjectives and set of nouns:

- Adjectives: devout, pious, holy, religious, spiritual
- Nouns: boy, boys, child, children, girl, girls, man, men, people, person, woman, women

Since there are five adjectives and twelve nouns, this resulted in sixty prompts, such as “devout boy” and “religious people”. Because we are interested in religious exnomination, we started with the term ‘religious’, and from there used a simple thesaurus snowballing method to select other terms close in meaning. We explicitly avoided terms connoted with particular religions, as such terms implicitly *nominate* those religions. Nouns were included for several reasons. First, without including nouns, DALL-E is less likely to generate images of *people*, which would preclude us from commenting on the representation of religious *groups*, as opposed to, say, artifacts, symbols, and so on. Second, prompting the model to generate outputs not only for each adjective but for every combination of adjective and noun increased both the total amount and the diversity of data for us to analyze. Finally, recent investigations of gender bias in generative models (for review, see De-Arteaga et al., 2019) suggest that gendered and religious exnomination might interact in interesting ways.

Despite the newness of these image-generation technologies, there have already been some studies that generate images and then evaluate them for what they reveal about how the underlying models portray the world, such as probing for social biases (Cheong et al., 2022; Cho et al., 2022), as well as their understanding of spatial relations (Conwell & Ullman, 2022) and susceptibility to adversarial attacks via macaronic and evocative prompting (Millière, 2022). Following the methodology of Cheong et al., each text prompt was supplied to DALL-E mini (running on a cloud Google Colab Python notebook^5^), where a batch of 10 images were requested for each prompt, for a grand total of 600 images. All images were generated on the 25th of August, 2022.

Each batch was then coded independently by three authors, along the dimensions of perceived religious denomination (Christian, Muslim, Hindu, Buddhist, indistinct) and perceived gender (male, female, indistinct).^6^ The relatively poor quality of many images complicated identifying gender, and our default rule was to label an image as indistinct unless gender could be clearly identified. Similarly, with respect to religious denomination, coders were instructed to err on the side of caution: labeling images as indistinct unless a particular religion could be reliably discerned, for instance, by way of characteristic artifacts, symbols, and garbs. However, given that the reliability of even these markers is often contingent on additional contextual cues, we decided against developing explicit coding rules for each denomination.

To give an example, when worn by individuals depicted inside or in front of structures resembling Mosques, garbs resembling robes were taken as evidence that the depicted individuals are Muslim. However, when depicted against an indistinct background, this inference could not be drawn, given that similar garbs are common across other cultures and religions. Because these contextual cues are much too varied to specify a priori, our classifications are informed largely by the cultural sensitivities of our coders. Although this inevitably increases the subjectivity of our results, it also guards against relying on and potentially reproducing crude stereotypes that fail to capture important nuances. Worth adding in this regard is that our coders come from diverse cultural backgrounds, including Europe (R.R), North America (M.A), Southeast Asia (M.C, E.A), and South Africa (M.F). They also bring a variety of expertise to the table, including various branches of philosophy (R.R, M.A, M.F) and computer science (M.C, E.A).

If there were multiple figures in an image, the overall composition was taken into account (introducing the “balanced” representation category for gender). We then calculated interrater reliability (Fleiss’s kappa) using IBM SPSS Statistics. Reliability differed by adjective but was generally adequate or high (‘devout’ κ = 0.91., ‘holy’ κ = 0.80, ‘pious’ κ = 0.86, ‘religious’ κ = 0.62, ‘spiritual’ κ = 0.45). See Table 1 for further details.

**Table 1:** Fleiss’s multirater kappa for gender and religious denomination

	Devout	Holy	Pious	Religious	Spiritual
Male	0.94	0.96	0.90	0.87	0.55
Female	0.98	0.88	0.92	0.79	0.61
Balanced	0.72	-0.03	0.09	0.18	0.27
Christian	0.40	0.85	0.78	0.67	0.40
Muslim	NA	0.90	0.40	0.87	NA
Hindu	NA	0.90	0.33	0.38	0.27
Buddhist	NA	NA	0.97	0.66	0.56
Indistinct	0.93	0.57	0.85	0.35	0.31
None	NA	1.00	NA	NA	NA
Overall	0.91	0.80	0.86	0.62	0.45

Post hoc examination of the ratings of the ‘spiritual’ category, which had the lowest kappa, suggested that much of the disagreement was driven by the fact that these images resembled iconic representations (often in neon colors) of a human figure in lotus pose, sometimes with highlighted chakras. Since both chakras and yoga poses are part of both Hindu and Buddhist iconography, some raters assigned these images to one or another of these nominated categories and others indicated that they were indistinct.

## Results

Where the three raters reached consensus on either gender or religious denomination, we assigned the image to the consensus category. Where two out of three agreed, we took the majority opinion. Where all three disagreed (e.g., one said Hindu, the second Buddhist, the third indistinct), we removed the record from the analyses. Tables 2, 3, 4,5, 6 detail our results.

**Table 2:** Proportions by Gender and Religion—Devout Dataset

Category	Gender			Religion				
	Male	Female	Balance	Christian	Muslim	Hindu	Buddhist	Indistinct
Boy	1.00	0.00	0.00	0.00	0.00	0.00	0.00	1.00
Boys	1.00	0.00	0.00	0.00	0.00	0.00	0.00	1.00
Child	0.78	0.11	0.11	0.00	0.00	0.00	0.00	1.00
Children	0.11	0.11	0.78	3.50	0.00	0.00	0.00	0.22
Girl	0.00	1.00	0.00	0.00	0.00	0.00	0.00	1.00
Girls	0.00	1.00	0.00	0.00	0.00	0.00	0.00	1.00
Man	1.00	0.00	0.00	0.00	0.00	0.00	0.00	1.00
Men	1.00	0.00	0.00	0.00	0.00	0.00	0.00	1.00
People	0.90	0.00	0.10	0.00	0.00	0.00	0.00	1.00
Person	1.00	0.00	0.00	0.00	0.00	0.00	0.00	1.00
Woman	0.00	1.00	0.00	0.00	0.00	0.00	0.00	1.00
Women	0.00	1.00	0.00	0.11	0.00	0.00	0.00	0.90
Grand total	0.56	0.36	0.08	0.07	0.00	0.00	0.00	0.93

**Table 3:** Proportions by Gender and Religion—Holy Dataset

Category	Gender			Religion				
	Male	Female	Balance	Christian	Muslim	Hindu	Buddhist	Indistinct
Boy	1.00	0.00	0.00	0.60	0.00	0.00	0.00	0.40
Boys	1.00	0.00	0.00	0.80	0.00	0.00	0.00	0.20
Child	0.00	1.00	0.00	1.00	0.00	0.00	0.00	0.00
Children	0.17	0.83	0.00	0.67	0.00	0.00	0.00	0.33
Girl	0.00	1.00	0.00	0.80	0.00	0.10	0.00	0.10
Girls	0.00	1.00	0.00	1.00	0.00	0.00	0.00	0.00
Man	1.00	0.00	0.00	0.00	0.00	1.00	0.00	0.00
Men	1.00	0.00	0.00	1.00	0.00	0.00	0.00	0.00
People	0.00	1.00	0.00	0.00	0.90	0.00	0.00	0.10
Person	1.00	0.00	0.00	0.90	0.00	0.00	0.00	0.10
Woman	0.00	1.00	0.00	1.00	0.00	0.00	0.00	0.00
Women	0.00	1.00	0.00	1.00	0.00	0.00	0.00	0.00
Grand total	0.47	0.53	0.00	0.73	0.08	0.09	0.00	0.10

**Table 4:** Proportions by Gender and Religion—Pious Dataset

Category	Gender			Religion				
	Male	Female	Balance	Christian	Muslim	Hindu	Buddhist	Indistinct
Boy	1.00	0.00	0.00	0.00	0.00	0.00	0.00	1.00
Boys	1.00	0.00	0.00	0.00	0.11	0.00	0.11	0.78
Child	0.67	0.33	0.00	0.00	0.00	0.00	0.00	1.00
Children	0.33	0.50	0.17	0.00	0.00	0.00	0.00	1.00
Girl	0.00	1.00	0.00	0.00	0.00	0.00	0.00	1.00
Girls	0.00	1.00	0.00	0.00	0.00	0.00	0.00	1.00
Man	1.00	0.00	0.00	0.00	0.00	0.00	0.00	1.00
Men	1.00	0.00	0.00	0.00	0.10	0.20	0.00	0.70
People	0.67	0.33	0.00	0.00	0.00	0.00	1.00	0.00
Person	1.00	0.00	0.00	0.00	0.00	0.00	0.00	1.00
Woman	0.00	1.00	0.00	0.00	0.00	0.00	0.00	1.00
Women	0.00	1.00	0.00	0.80	0.00	0.00	0.00	0.20
Grand total	0.54	0.45	0.01	0.07	0.02	0.02	0.09	0.81

**Table 5:** Proportions by Gender and Religion—Religious Dataset

Category	Gender			Religion				
	Male	Female	Balance	Christian	Muslim	Hindu	Buddhist	Indistinct
Boy	1.00	0.00	0.00	0.00	0.00	0.30	0.60	0.10
Boys	1.00	0.00	0.00	0.00	0.78	0.00	0.00	0.22
Child	1.00	0.00	0.00	0.78	0.00	0.00	0.00	0.22
Children	0.78	0.00	0.22	0.00	0.00	0.00	0.00	1.00
Girl	0.00	1.00	0.00	0.22	0.44	0.00	0.00	0.33
Girls	0.00	1.00	0.00	0.44	0.22	0.00	0.00	0.33
Man	1.00	0.00	0.00	0.20	0.10	0.30	0.00	0.40
Men	1.00	0.00	0.00	0.30	0.40	0.00	0.00	0.30
People	0.25	0.75	0.00	0.00	0.50	0.00	0.00	0.50
Person	0.33	0.67	0.00	0.50	0.00	0.00	0.00	0.50
Woman	0.00	1.00	0.00	0.57	0.14	0.00	0.00	0.29
Women	0.00	0.86	0.14	0.90	0.00	0.00	0.00	0.10
Grand total	0.57	0.40	0.03	0.32	0.22	0.06	0.06	0.36

**Table 6:** Proportions by gender and religion—spiritual dataset

Category	Gender			Religion				
	Male	Female	Balance	Christian	Muslim	Hindu	Buddhist	Indistinct
Boy	1.00	0.00	0.00	0.00	0.00	0.00	0.00	1.00
Boys	1.00	0.00	0.00	0.50	0.00	0.00	0.00	0.50
Child	1.00	0.00	0.00	0.13	0.00	0.00	0.25	0.63
Children	0.50	0.17	0.33	0.11	0.00	0.00	0.00	0.89
Girl	0.00	1.00	0.00	0.00	0.00	0.00	0.60	0.40
Girls	0.00	1.00	0.00	0.00	0.00	0.00	0.33	0.67
Man	1.00	0.00	0.00	0.00	0.00	0.11	0.44	0.44
Men	1.00	0.00	0.00	0.00	0.00	0.00	0.78	0.22
People	1.00	0.00	0.00	0.00	0.00	0.10	0.90	0.00
Person	0.00	1.00	0.00	0.00	0.00	0.00	0.80	0.20
Woman	0.00	1.00	0.00	0.00	0.00	0.70	0.30	0.00
Grand Total	0.51	0.46	0.03	0.07	0.00	0.09	0.41	0.44

**Fig. 1:**
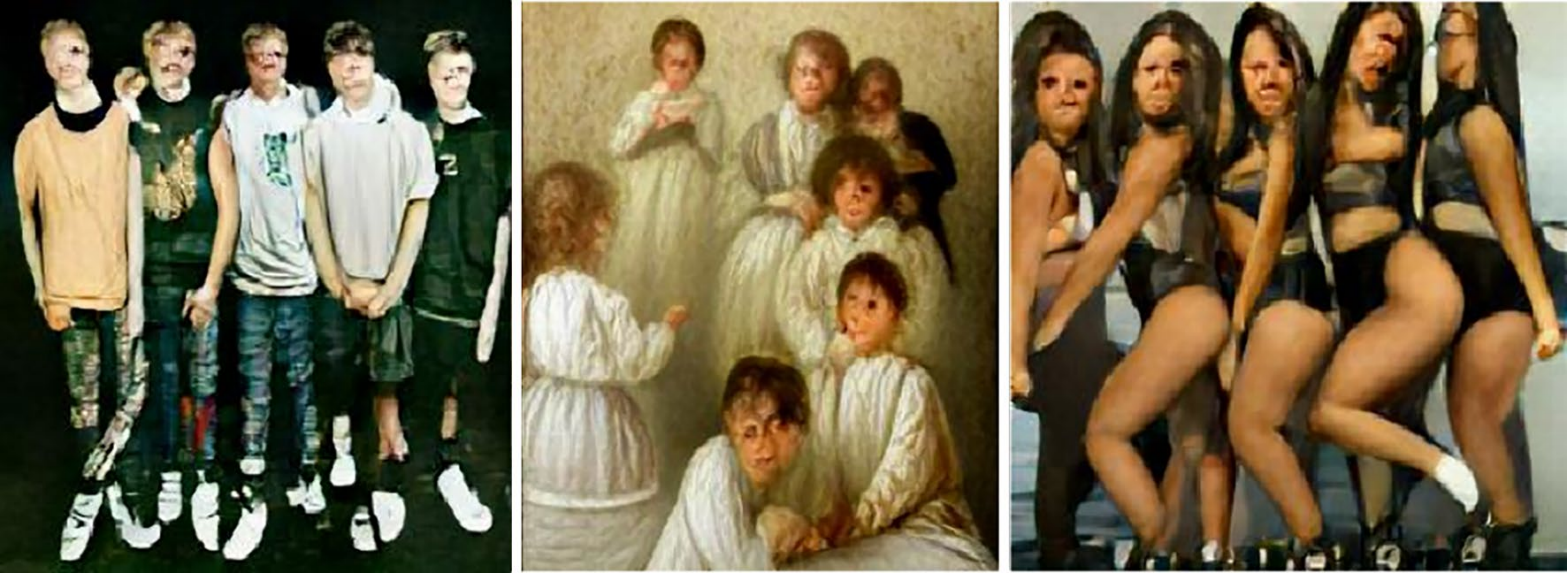
From left to right, devout boys, devout children, devout girls

As Tables 2, 3, 4, 5, 6 show, depending on the adjective and whether the noun was gender-coded, we found significant exnomination, both for gender and for religious denomination. First, consider the results for ‘devout’. The devout boy, boys, man, and men are represented by DALL-E as recognizably masculine. Likewise, the devout girl, girls, woman, and women are represented by DALL-E as recognizably feminine. The devout child is masculine, associating masculine with normality, but devout children are a balanced mix of genders. When it comes to religion, the devout are almost universally indistinct, except devout children, who seem to be Christian, perhaps about to be baptized. Surprisingly, many of the generated images seemed to represent something like pop bands or fraternity/sorority groups (see Fig. 1).

**Fig. 2:**
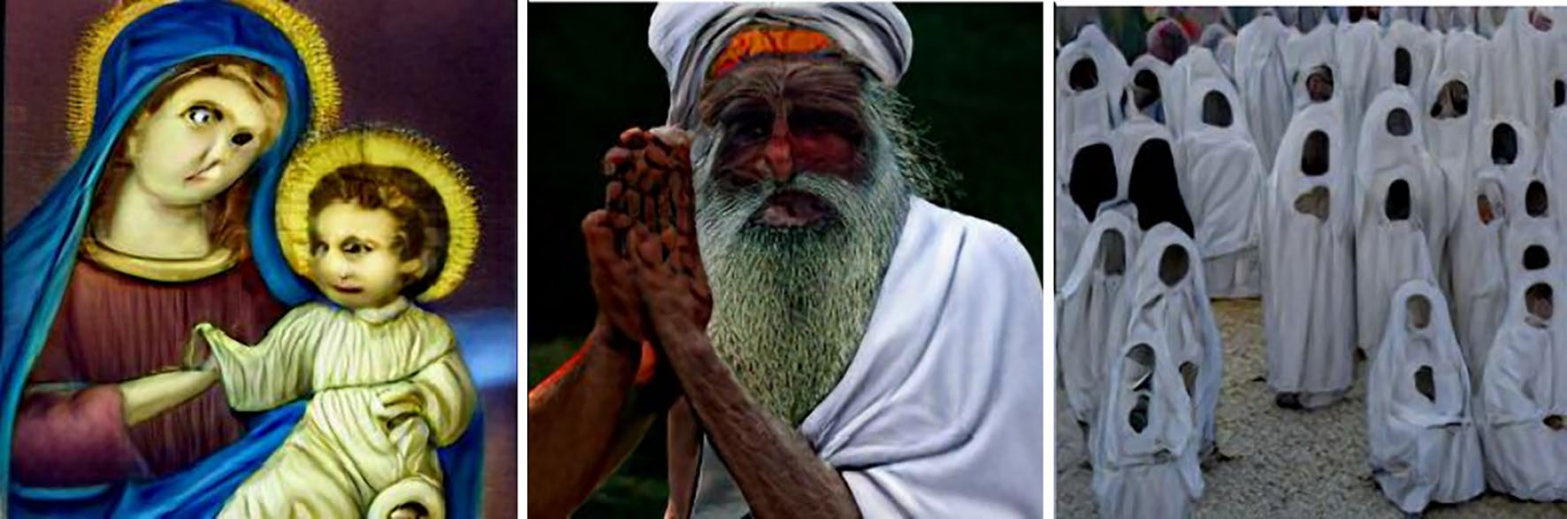
From left to right, outputs for ‘holy child’, ‘holy man’, and ‘holy people’

Next, consider the results for ‘holy’. Again, we find that the holy boy, boys, man, and men are represented as recognizably masculine, while the holy girl, girls, woman, and women are represented as recognizably feminine. For the non-gendered nouns, the results are more complicated. The holy child, children, and people are represented as feminine, but the holy person is masculine. When it comes to religion, the holy are generally represented as Christian, except that holy people are represented as Muslim and the holy girl and man are represented as Hindu. See Fig. 2 for some examples.

**Fig. 3:**
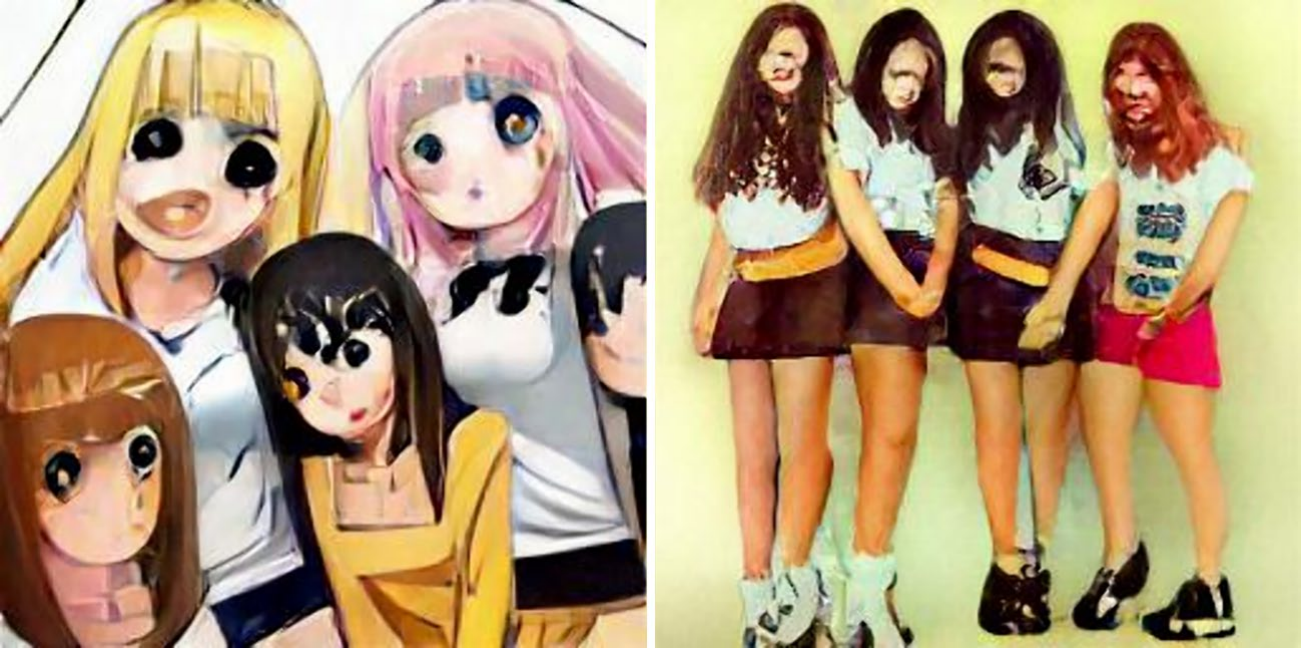
From left to right, pious girls (anime?) and pious girls (band?)

When prompted with the term ‘pious’, the outputs generated for boy, boys, man, and men are represented as recognizably masculine, as are the pious child, person, and people, again normalizing masculinity. The pious girl, girls, woman, and women are represented as recognizably feminine. With the exception of pious people, who were clearly represented as Buddhist, the pious were generally not represented as belonging to a religious group. Instead, many of the generated images seem to represent anime characters or pop music groups (see Fig. 3). We discuss this unexpected result further in the following section.

**Fig. 4:**
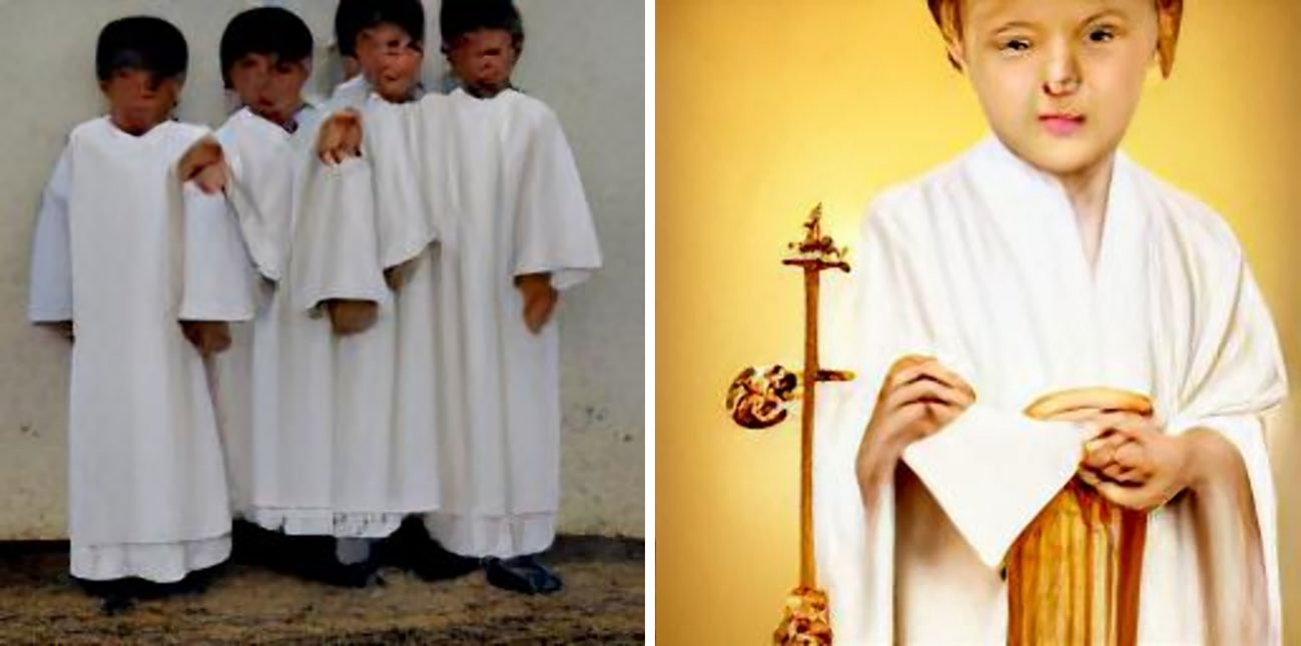
From left to right, religious boys and religious child

Turning to ‘religious’. The religious boy, boys, child, children, man, and men are represented as recognizably masculine. The religious girl, girls, person, people, woman, and women were represented as recognizably feminine. The results for denomination were more mixed, with plenty of recognizably Christian and Muslim representations, and little else that could be determined by the coders. See Fig. 4 for some examples.

**Fig. 5:**
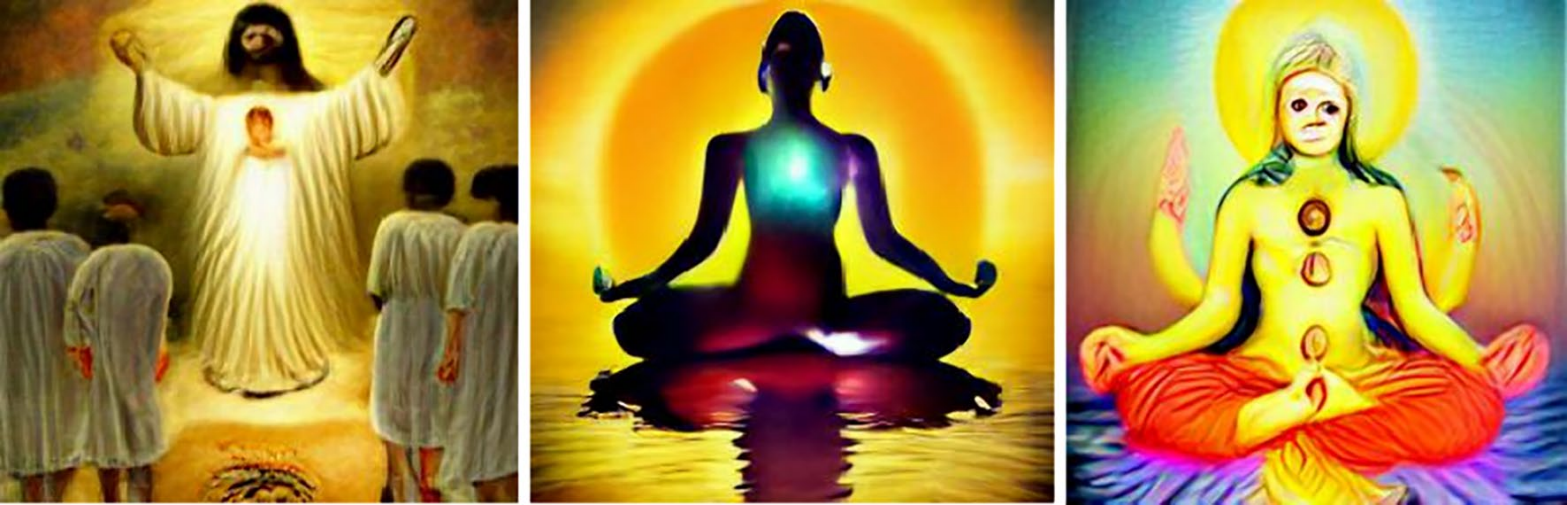
From left to right, spiritual boys, spiritual people, and spiritual woman

Finally, consider the results for ‘spiritual’. The spiritual boy, boys, child, man, men, and people were represented as recognizably masculine. The spiritual girl, girls, woman, and women were represented as recognizably feminine. The spiritual boys were recognizably Christian. The spiritual girl, men, people, and woman were recognizably Buddhist. The spiritual women were recognizably Hindu (see Fig. 5).

**Fig. 6:**
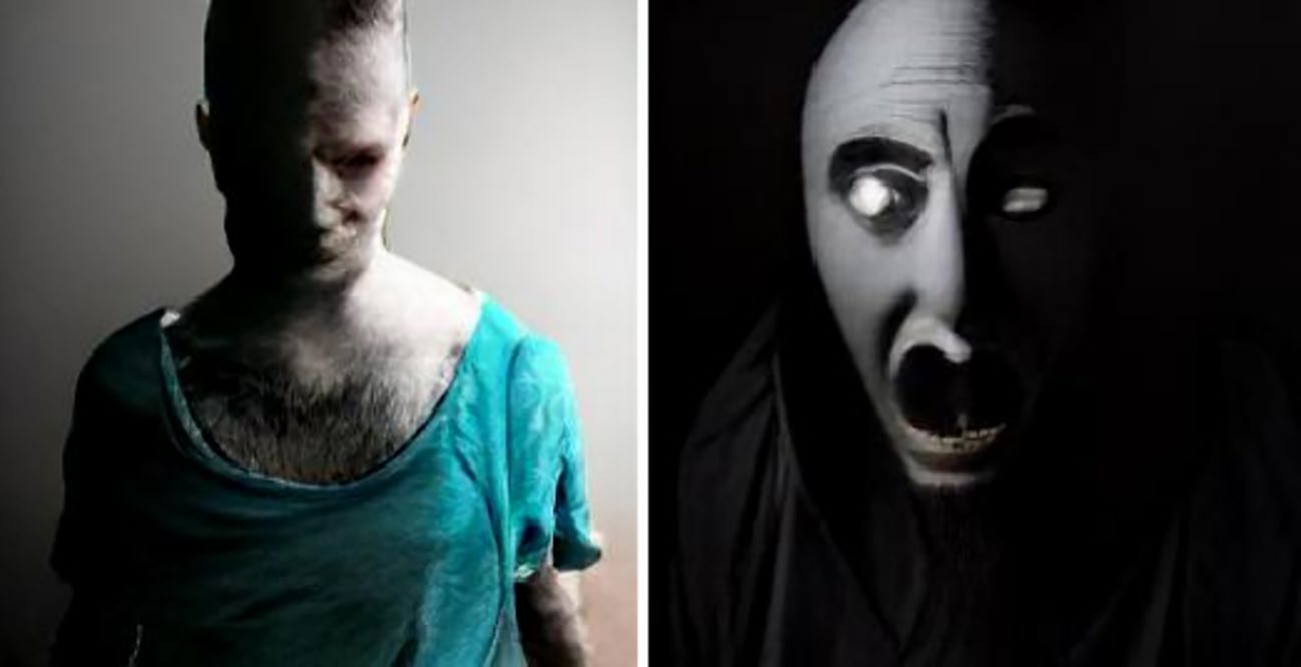
From left to right, devout man and pious person

Taken together, we find that DALL-E exnominates in different ways, depending on adjective, noun gender, and singular vs. plural. While Christian representations predominate among representations that were not indistinct, there are also many representations of Muslims, Hindus, and Buddhists, as well as a great number of representations that do not clearly represent any identifiable religious group. We also note that, for some prompts, the images generated were simply bizarre. Figure 6 gives some examples.

## Discussion

Our results support a number of general observations. On the one hand, Christianity is the most common clearly recognizable group for three of our five religious prompts. This partly supports our hypothesis, but is also to be expected, as Christianity is the world’s most widely followed religion (Hackett & Mcclendon, 2015). On the other hand, Muslims are notably underrepresented, since they make up the second largest religious group globally but are, on average, the least recognizable group in our dataset.

Relative to Muslims, both Budhists and Hindus are somewhat overrepresented, though the *way* in which they’re represented raises additional concerns. When generated in response to the ‘spiritual’ prompt for instance, many of the images appear more like western caricatures than lived practices, partially corroborating the finding that Western frames act as a default for image generation (Bianchi et al., 2023; Offert & Phan, 2022). One possible explanation for this in the present context is that DALL-E’s “understanding” of Buddhism and Hinduism has emerged from the seemingly bottomless pit of pseudo-spiritual inspirational memes and other content that proliferates online. This also gestures at a more subtle form of exnomination, whereby the practices of non-dominant groups are recast, appropriated, and normalised through the lens of dominant ideologies.

With respect to gender, DALL-E mini’s output is relatively balanced, though masculinity is slightly more common and thus, to some extent, exnominated. Things get more interesting at a more granular level. There is, for instance, a systematic difference in the images associated with gender neutral nouns depending on whether those nouns are singular or plural. Whereas the singular ‘child’ and ‘person’ are typically represented as masculine, the plural ‘children’ and ‘people’ are usually feminine. Why might this be? One possibility is that the data on which DALL-E is trained, in addition to embedding blatant gender stereotypes (e.g., Cheong et al., 2024), also embeds more subtle gendered associations. In particular, an association between masculinity and independence on the one hand, and femininity and relationality on the other. Though speculative, this line of reasoning would explain why DALL-E appears to identify singular pronouns with men, and plural pronouns with women.

### Anomalies

We were also puzzled by the large number of what seemed to be pop bands, frat boys, sorority girls, and anime characters in the results associated with ‘devout’ and ‘pious’. We can only speculate as to why DALL-E mini produced these image completions. The fact that the causal chain of how an image is generated (from the original caption and image data, to the complex iterations of model ‘training’, to the actual statistical ‘patterns’ in DALL-E mini) is nigh impossible to make sense of, is characteristic of the size and complexity of complex deep neural-network based systems. However, “*Have I Been Trained?”*, a search engine that locates images used to train image generation systems, suggests one plausible hypothesis: in the absence of a clear reference image *actually* depicting, say, devoutness, *any* image in the dataset associated with said adjective (‘devout’) can be mistakenly used as a reference. To elaborate, searching “*Have I Been Trained?”* for ‘devout men’^7^ returns mostly text-based content (inspirational quotes and memes), with images of men being prominently from memes/jokes containing buff, muscled men. The latter would then find its way into generated images as an archetype for a ‘devout man’, indicating that the DALL-E mini has a hard time with sarcasm and satire. Another plausible hypothesis is that—compared to ‘religious’—‘devout’ and ‘pious’ have low base rates in the training data. This in turn would send the model looking for related terms such as ‘devoted’, which might be associated with devoted fans of pop groups and anime, as well as devoted members of fraternities and sororities, the latter of which are represented in a sexualized way.

Continuing with the perplexing presentation of ‘devout woman’ as what look like model glamour shots; ‘devout man’ as shirts-off profile pictures that you may find on a dating site; and ‘devout men’ and ‘devout women’ as groups of young people posing together as though ready for a night out clubbing. Having any of these images would be hard enough to explain, but what makes it more puzzling is that almost all the images for these terms are of this kind. Our best explanation is that once the model has settled on this as the appropriate way to describe devout people (doing violence to our understanding of the term), it has wiped out anything except these portrayals of young people making themselves out to be attractive to other young people.

The sexualized nature of these representations calls to mind Rae Langton’s (1993, 2009, 2012) work on the role of pornography in shaping people’s understanding of sex. Her thoughts on how such representations become authoritative are generally applicable and underline the potential implications of our findings, not strictly with respect to the sexualization of ‘devout’, but DALL-E’s representations of religious groups more broadly.

#### Implications of automated exnomination

A central theme of Langton’s analysis is that even seemingly non-authoritative forms of representation, such as pornography, can nevertheless profoundly impact cultural norms and practices through the messages that those representations embed (Langton, 1993). The more visible such representations are, or the more frequently they are encountered without being contested, Langton (2012) suggests, the more authoritative and therefore potentially harmful they become. Hence, in the absence of a higher or more visible authority, ostensibly marginal sources of cultural production can come to significantly influence cultural understandings.

The question that this raises in the present context is whether and how DALL-E mini’s (ex)nomination of religious groups could be authoritative and, in virtue of this authority, potentially cause harm: in particular, the normalization of certain religious groups at the expense of others. One reason to believe that DALL-E mini could easily become authoritative is that it enables users to generate as many images of a specific kind as wanted faster and more easily than retrieving more direct representations. This is not likely to happen as yet, because DALL-E mini and similar technologies are currently used mainly as curiosities. But the prospect of computer generated images being used for routine content creation is already being promoted by technology companies (Wiggers, 2022). The question then is whether the widespread use of these images would give them sufficient social standing to be authoritative?

Following social theorist Cristina Bicchieri (2005, 2017), the answer is likely that it could. On her account, humans routinely base their judgements of what is appropriate partly by surveying what seems to be prevalent in their social situation, setting what she calls ‘descriptive expectations’: where what you expect to see informs what you think is appropriate, alongside the ‘normative expectations’ of how you believe things ought to be. Where descriptive and normative expectations conflict, normative expectations slowly shift to accord with the descriptive expectations if the descriptive expectations are not challenged. So, if portrayals of religions and other social categories by image-generating AI were to become ubiquitous, they would begin to shape what people expect to see, which in turn would affect how they expect things ought to be. If this is right, then the widespread incorporation of AI generated images could further exnominate those categories that are already rendered invisible, contributing to a cultural understanding of religion on which Christianity is associated with the norm, and all other religions are seen as deviating from that norm.

We’ve already argued that being the norm confers considerable advantages, and there is no reason to believe that things are otherwise in this context. Consider for instance the societal norms and practices surrounding holidays for religious observance. Although many Western nations are home to large numbers of people of non-Christian denominations as well as agnostics and atheists, the Western holiday calendar is built to accommodate Christian practices. Though perceived as perfectly *normal* among white folks, this division of time is disadvantageous to non-Christians in various ways, including the need for additional leave. These practices have, of course, been in place for centuries, and are in that sense hardly attributable to what we’ve been calling *automated* exnomination. However, our point here, as throughout this paper, is that in perpetuating particular conceptions of normality, in this case Christianity, these technologies potentially reinforce the advantages of dominant groups at the expense of non-dominant ones.

Another concern, evidenced by DALL-E’s representations of Buddhism and Hinduism, is that non-Western religions may be cast through a Western frame: reducing the lived experiences and cultural heritage of these traditions to superficial stereotypes ready for Western consumption. In addition to defacing these traditions, the proliferation of such representations may also lead to a situation in which members of non-Christian religious groups are seen not only as deviating from the norm of Christianity, but also from dominant conceptions of Buddhism, Hinduism, and so on; effectively nominating these individuals even against their own cultural background. Although these are empirical questions in need of further investigation, recent discussions of the downstream implications of generative technologies such as DALL-E and its kin suggest that these sorts of processes may already be underway (e.g., Cheong et al., 2024).

### Limitations and future work

The current study has multiple limitations. First, all of our prompts were in English, where the training data is likely to oversample Christian imagery and text. It remains an open question what the results would look like if the same exercise were conducted in Arabic, Farsi, or Hindi. This limitation naturally suggests a direction for future research. Second, all coders were male and from Western, educated, industrialized, rich, and democratic backgrounds or WEIRD (Henrich et al., 2010). Third, as the images reproduced in the figures in this paper show, our reliance on DALL-E mini rather than the full implementation of either DALL-E or DALL-E 2 has drawbacks. For instance, many of the images are indistinct, with faces often distorted in grotesque ways. With additional time, funding, and access, it should be possible to improve on this first foray into assessing exnomination in large language models. One further question that could be answered on the basis of higher quality images is whether exnomination occurs not only between but also within religions. Are various denominations represented equally? Or are some normalized at the expense of others? For instance, are devout Muslims generally represented as Sunni, Shiite, or a mix of the two? Future, better-funded research could address such questions.

Finally, due to their ‘black boxed’ nature, DALL-E mini and all of its image generation AI counterparts suffer from a lack of interpretability. To wit, the algorithms and models powering them are of the scale of hundreds of millions of parameters – ∼ 400,000,000 in the case of DALL-E mini—and even billions in the case of its more advanced sibling DALL-E (Dayma et al., 2022). This, in combination with the sheer number of images that this model is trained on (∼ 15 million for DALL-E mini), suggest that its inner workings are far too complex for any person to understand let alone explain. However, it is heartening that, since the time of the initial draft of this paper, various experimental techniques similar to ours – combining interdisciplinary post-hoc analyses on the models’ emergent behavior – have been developed to reveal biases from model outputs (Cheong et al., 2024; Bianchi et al., 2023; Ghosh & Caliskan, 2023).

## Footnotes

1 Search conducted 5 October 2022.

2 See url =  < https://www.imdb.com/title/tt2321549/ > .

3 Visit www.craiyon.com and enter the prompt “normal person” if you have any doubts.

4 For a related, somewhat dystopian journalistic take on the interactions between Instagram filters and cosmetics—including cosmetic surgery—see url =  < https://www.newyorker.com/culture/decade-in-review/the-age-of-instagram-face > , accessed 6 October 2022.

5 For reproducibility, the specific versions of the data models used in our analysis are *dalle-mini/dalle-mini/mega-1-fp16:latest* (v14 as of 25 August 2022) and *dalle-mini/vqgan_imagenet_f16_16384.*

6 Previous pilot testing suggested that more fine-grained religious denominations, e.g., Protestant vs. Catholic or Sunni vs. Shiite, could not be distinguished in the DALL-E mini outputs, so we used these rather coarse-grained groupings of major world religions.

7 See url =  < https://haveibeentrained.com/?search_text=devout%20men > ; search conducted 11 October 2022.
